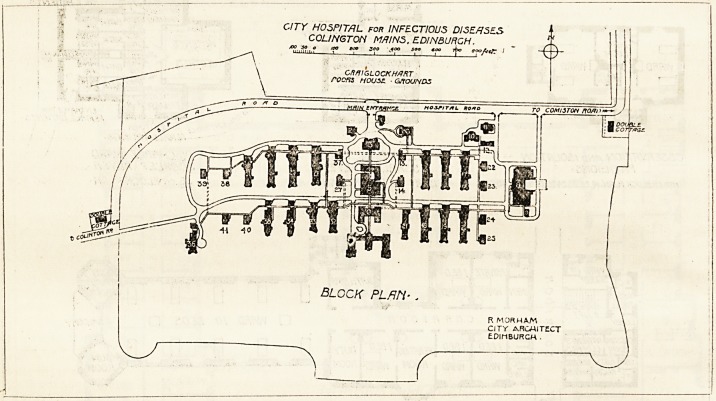# The City Hospital for Infectious Fevers, Edinburgh

**Published:** 1905-06-17

**Authors:** 


### HOSPITAL ADMINISTRATION.

### CONSTRUCTION AND ECONOMICS.

### THE CITY HOSPITAL FOR INFECTIOUS FEVERS, EDINBURGH.

Up to the year 1885 the Corporation of the City of Edinburgh possessed no means of dealing with outbreaks of infectious fevers other than that afforded by the voluntary co-operation of the governing body of the Royal Infirmary. The old buildings of the last-named institution had, since the erection of the new infirmary in Lauriston, been used for the reception of fever cases, but still remained a part of the infirmary and were maintained by the funds of the charity. This arrangement ceased in 1885 when the Corporation assumed the responsibility which properly belonged to them and took over the buildings. These old buildings which had become unsuitable and inadequate to the needs of the infirmary were no better suited to the purposes of a fever hospital. The rapidly increasing use made of the hospital also, and the lesson taught by an outbreak of small-pox in 1894, convinced the City authorities of the necessity for providing a hospital adequate to the needs of the population. The use of the site of the old hospital being for sufficient reasons decided against, an estate of about 300 acres at Colinton Mains on the outskirts of the city was acquired, and about 40 acres set apart for the purpose of the new fever hospital. The site is an excellent one in every respect. Situate on high ground, about 400 feet above sea level, well outside the city, it has an extensive view over the Pentland Hills towards the south, while it is well sheltered from the north. In point of area the proportion of site to patient is 1 acre to 15 patients, a wisely liberal allowance.

A point that at once strikes the visitor as unusual is the number of different diseases which are here provided for. To anyone accustomed to the southern methods of dealing with infectious fevers, by which, as a rule, three diseases—scarlatina, typhoid, and diphtheria, with occasionally a fourth, typhus, only are recognised—it will come as a surprise to find definite provision made for measles, whooping cough, chicken-pox, and erysipelas. The total provision for patients amounts to 600 beds, and these are distributed as follows:—Scarlatina, 320; typhoid, 76; diphtheria, 30; measles, 76; typhus, 10; erysipelas, 30; whooping cough, 19 chicken-pox, 19; isolation, 16; observation, 4. Total, 600.

The whole of the ward blocks, except that for typhus, the observation wards, and six out of the eight isolation blocks are connected together with the general administration buildings by covered ways open at the sides, and an ambulance roadway provides for wheeled access to all the blocks. The pavilion for scarlatina, typhoid, diphtheria, measles, and erysipelas, are two storeys in height, the remainder being one storey only.

At the main entrance, which, it should be noted, is on a private road some considerable distance from the Colinton Road, the nearest public highway, is the porter's lodge on one side, and the medical superintendent's house on the

SCARLET FEVER PAVILIONS·

Nos 15-20 ON BLOCK PLAN·

OBSERVATION AND ISOLATION PAVILIONS·

Nos ON BLOCK PLAN. 14.22.23.24.25.27.33.39.40.41.

RECEIVING WARD SCARLET FEVER·

13 & 37 ON BLOCK PLAN.

DISCHARGE WARD SCARLET FEVER·

26 ON BLOCK·PLAN·

DIPHTHERIA PAVILION·

28 ON BLOCK·PLAN.

WHOOPING COUGH AND CHICKEN POX PAVILIONS.

34 AND 35 ON BLOCK PLAN.

other; while immediately facing is the block of general offices in which are the offices for the medical superintendent and matron and the quarters for the assistant medical staff. This building faces due north; going southward the buildings immediately behind the office block are stores, kitchen block, servants' home, and nurses' home. On the east side of this central group are placed the wards for scarlatina, and on the west side the wards for the other diseases. In the north-east corner of the site is a group of buildings comprising the education block, museum, mortuary, workshop and ambulance building, and in the south-east corner is the laundry and the boiler house and disinfecting rooms. For the scarlatina patients two detached buildings are for reception and discharge. A single reception-block serves for the group of diseases other than scarlatina, while to each pavilion is provided a complete set of discharge-rooms. The pavilions for scarlatina contain a general ward for 20 beds, a separation ward for two beds, a private ward for one bed intended for paying patients, together with the usual offices. The sanitary offices for the general ward are placed in a tower projecting from the centre of the ward. The chief argument in favour of this position, as against the more usual one at the extreme end of the ward, is that the distance which has to be traversed, either by patient or nurse, from the furthest bed, is reduced by one-half; while the main objection to a projecting building on the side of the ward is that it must impede ventilation, and to some extent overshadow part of the ward. The gain in convenience of the central position is, we think, much more than counterbalanced by the loss of sunlight and free ventilation.

The position commonly occupied by the sanitary towers at the extreme end of the ward is taken by an emergency exit staircase on one side, and a "sun room" on the other.

The general wards are warmed by two sets of double fireplaces with descending flues, and by hot water radiators. The W.C. attached to the one-bed ward is separated from the ward by a lobby which is ventilated on one side only, and the disconnection between the W.C. attached to the two-bed ward is not as complete as it should be. We regret to have to draw attention to these faults in an otherwise excellent plan; but it cannot be too strongly insisted on that all sanitary offices belonging to wards must be properly disconnected. On the further side of the corridor which connects each pavilion with the covered way of communication is the staircase to the upper floor, ward scullery and pantry, orderly-room for brooms, etc., and a nurses' toilet-room with W.C. attached. The reception block for scarlatina patients contains a reception-room, examination-room, bathroom, blanket store, lavatory and W.C., with a room for the nurse with W.C. and lavatory attached. The discharge block is an excellently planned building and provides for the simultaneous discharge of two male and two female patients. Each set consists of an undressing-room, a bathroom and a dressing-room, the last-named opening into a waiting-room in which the friends or relations of the patients can wait. A general store for patients' disinfected clothes serves for both sexes. The discarded hospital clothes are deposited in a lobby adjoining the undressing-rooms, from whence they are removed by an outer door.

The pavilion for diphtheria is modelled on the same lines as those for scarlatina, the main difference being that the general ward is for a less number of beds (10), but gives to each bed 2,845 cubic feet instead of 2,000 feet, that it has five single-bed wards, and that it has an operation-room. The discharge rooms also are placed in the service block on the opposite side of the corridor.

The typhoid blocks differ slightly from the diphtheria. The general wards contain 16 beds, with a cubic space per bed of 2,514 feet. The ward offices follow those of the scarlatina blocks, with the addition of a set of discharging rooms at the back of the staircase.

The blocks for whooping-cough and chicken-pox are one storey only, and each contain two wards, one for eight, the other for ten beds, the cubic space in the one being 1,774 feet, in the other 1,859 feet per bed. One single-bed ward communicates directly with the ten-bed ward and with the duty room. The latter is central, and serves for both general wards. A small block separated from the main block by the covered way contains the discharge room and the nurses' toilet-room.

The measles blocks are planned on the same lines as the scarlatina blocks, with the addition of discharge rooms, as in the typhoid blocks. The cubic space in the general wards is 1,690 feet per bed.

The typhus blocks contain two wards for four beds each, with two small wards for one bed each, the usual offices, and a detached discharge block.

The observation and isolation pavilions each contain two wards for two beds each, with w.c. and sink-room properly disconnected in one case, but imperfectly so in the other, duty-room, linen-room and coal store, and space for moveable bath.

Of the official buildings there is not much to call for remark. The kitchen offices are admirably planned, and fitted with complete gas and steam cooking apparatus by Messrs. Slater and Co. Close to the kitchen are grouped the dining-rooms for nurses and servants, with the various stores and workrooms.

The Nurses' Home, which is four storeys high, provides accommodation for 130 nurses, with some spare rooms, three day-rooms and a library, two sick-rooms, a large recreation-room, and apartments for the matron. Luggage-lifts are provided in each wing and an adequate supply of bathrooms, lavatories, etc. To the south of the home an ample space of garden is set apart for the nurses' use.

The designs for this very complete and excellently arranged hospital were prepared by Mr. R. Morham, the City Architect, with the advice and co-operation of the Medical Officer of Health, Sir H. D. Littlejohn.

## Figures and Tables

**Figure f1:**
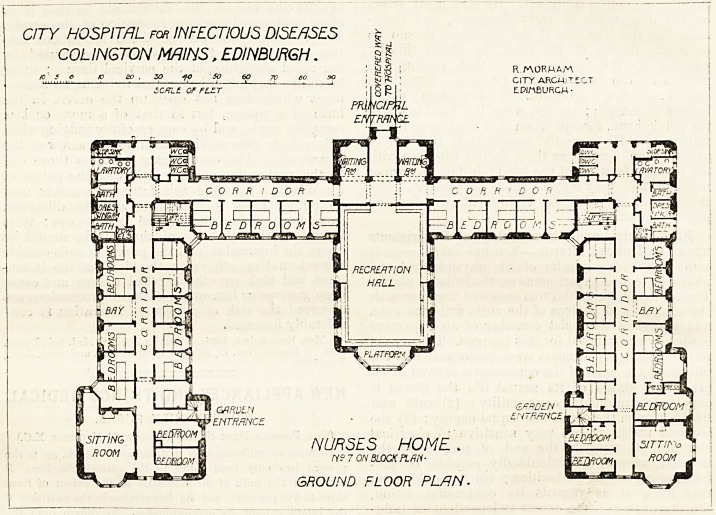


**Figure f2:**
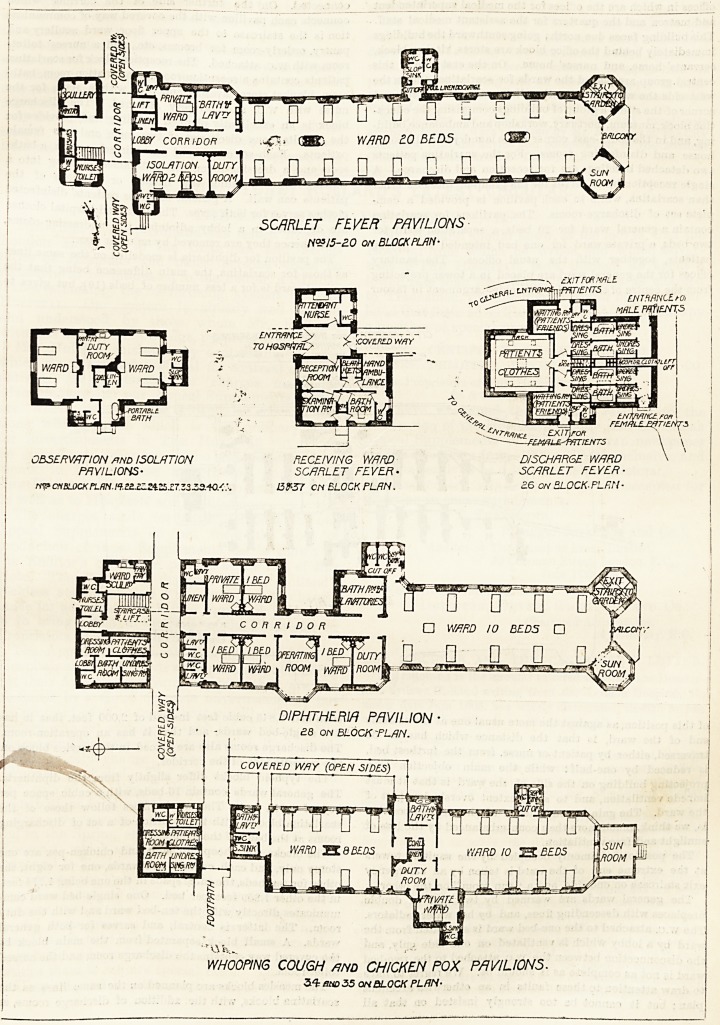


**Figure f3:**